# Supplementary figures and images for: SOFA score performs worse than age for predicting mortality in patients with COVID-19
Source: PLoS One. 2024 May 17;19(5):e0301013. doi: 10.1371/journal.pone.0301013 (PMC11101117; doi:10.1371/journal.pone.0301013)

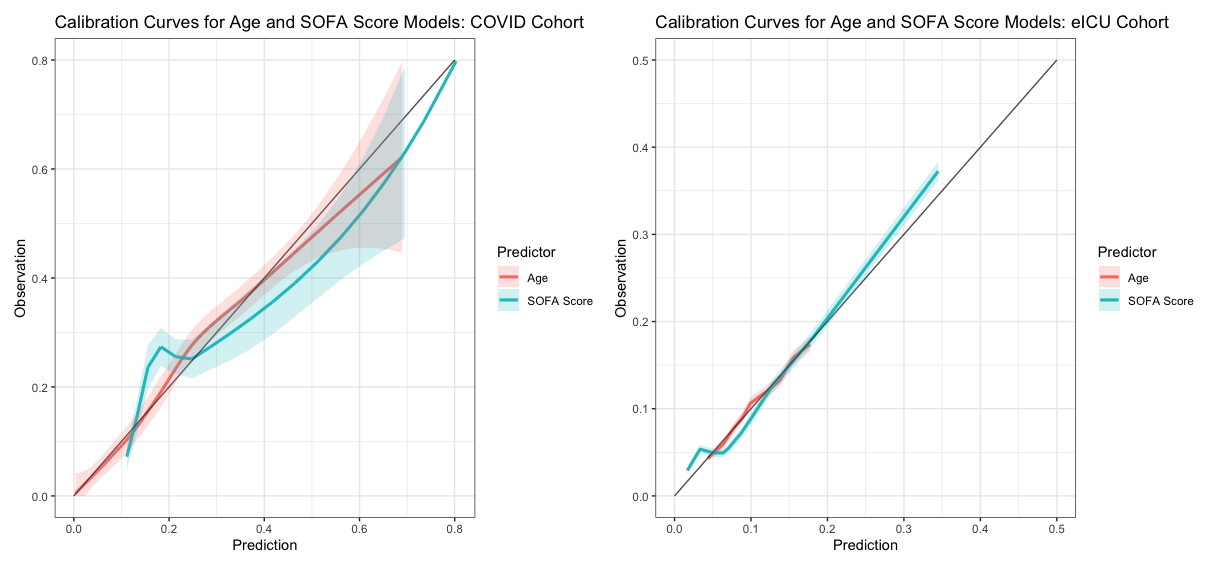

Supplement: S1 Fig — Calibration curves for age-based (red) and SOFA score-based (blue) models in both cohorts. The black line with a slope of 1 represents a perfectly calibrated model. (TIF) [file pone.0301013.s001.tif]

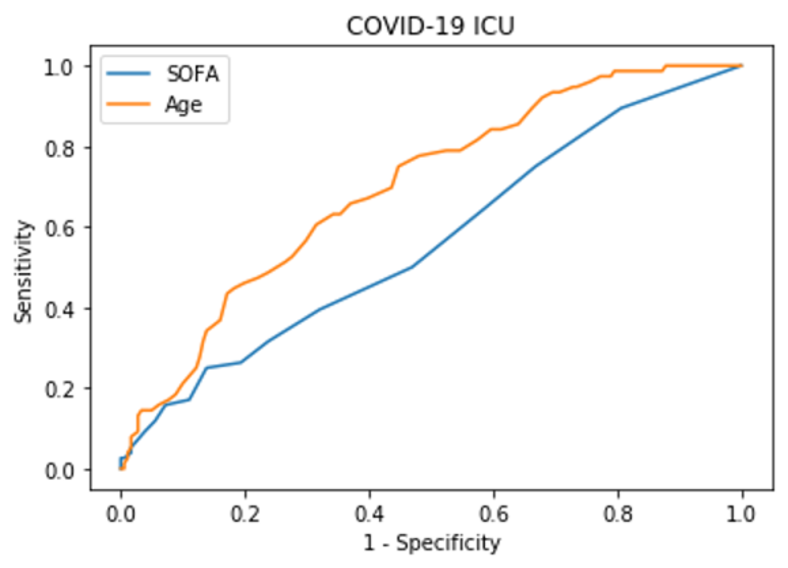

Supplement: S2 Fig — AU-ROC of age-based (orange) and SOFA score-based (blue) models calculated on subset of COVID-19 cohort that required ICU admission. (TIF) [file pone.0301013.s002.tif]

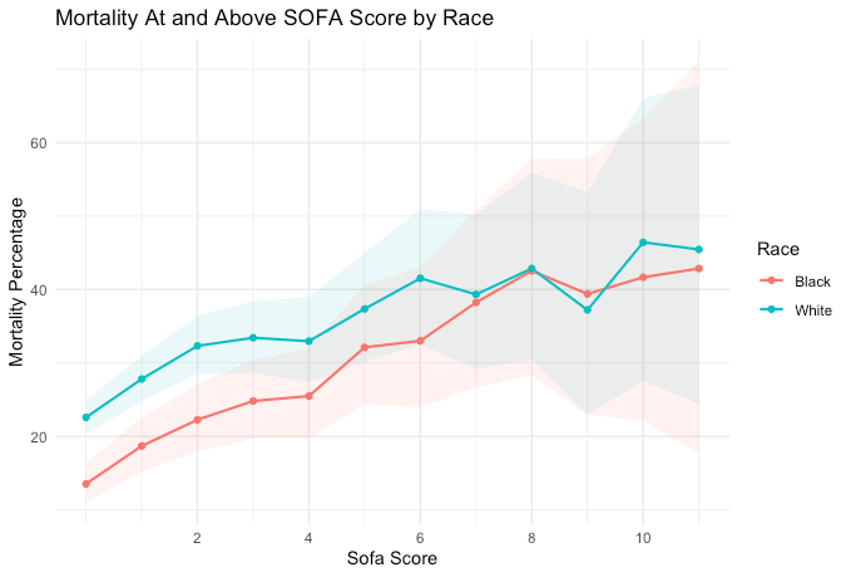

Supplement: S3 Fig — Mortality percentage at and above a given SOFA score in Black (red line) and White (blue line) patients in the COVID-19 cohort with 95% confidence intervals in shaded region. (TIF) [file pone.0301013.s003.tif]

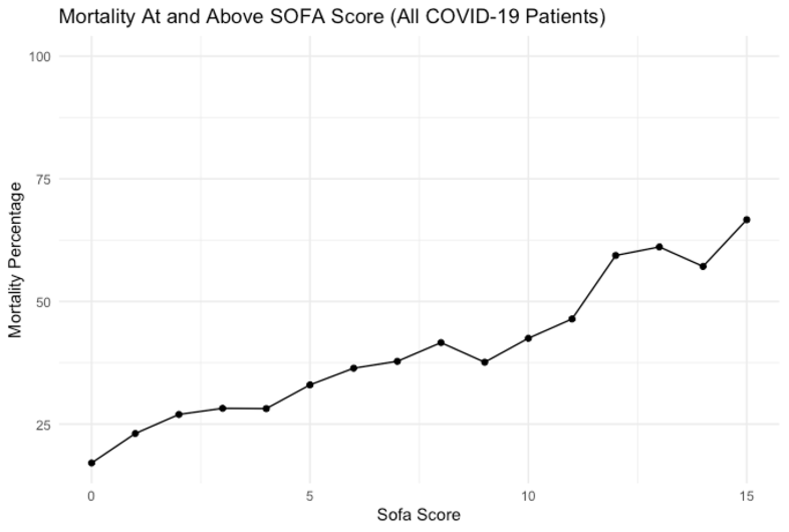

Supplement: S4 Fig — Mortality percentage at and above a given SOFA score for all patients in the COVID-19 cohort. (TIF) [file pone.0301013.s004.tif]
