# Supplementary material for: SOFA score performs worse than age for predicting mortality in patients with COVID-19
Source: PLoS One. 2024 May 17;19(5):e0301013. doi: 10.1371/journal.pone.0301013 (PMC11101117; doi:10.1371/journal.pone.0301013)
Supplement: Attachment — Submitted filename: Response to reviewers.docx [file pone.0301013.s006.docx]

Response to PLOS One Reviewer Comments

1/5/2023

We would like to thank the Academic Editor and Reviewers for their thoughtful comments and suggestions.

Our point-by-point response is formatted in *italics and red* below each comment for context

Response to Editor’s Comments

1. Please ensure that your manuscript meets PLOS ONE's style requirements, including those for file naming. The PLOS ONE style templates can be found at https://journals.plos.org/plosone/s/file?id=wjVg/PLOSOne_formatting_sample_main_body.pdf and https://journals.plos.org/plosone/s/file?id=ba62/PLOSOne_formatting_sample_title_authors_affiliations.pdf

*Thank you. We have updated the formatting of our manuscript to meet PLOS ONE’s style requirements.*

2. Note from Emily Chenette, Editor in Chief of PLOS ONE, and Iain Hrynaszkiewicz, Director of Open Research Solutions at PLOS: Did you know that depositing data in a repository is associated with up to a 25% citation advantage (https://doi.org/10.1371/journal.pone.0230416)? If you’ve not already done so, consider depositing your raw data in a repository to ensure your work is read, appreciated and cited by the largest possible audience. You’ll also earn an Accessible Data icon on your published paper if you deposit your data in any participating repository (https://plos.org/open-science/open-data/#accessible-data).

*Unfortunately, we are unable to publicly share the data from the COVID cohort (see data availability statement below. However, the eICU cohort is taken from the publicly available eICU dataset: Pollard, T., Johnson, A., Raffa, J., Celi, L. A., Badawi, O., & Mark, R. (2019). eICU Collaborative Research Database (version 2.0). PhysioNet. https://doi.org/10.13026/C2WM1R.*

3. We note that the grant information you provided in the ‘Funding Information’ and ‘Financial Disclosure’ sections do not match.

When you resubmit, please ensure that you provide the correct grant numbers for the awards you received for your study in the ‘Funding Information’ section.

4. Thank you for stating the following financial disclosure:

"The author(s) received no specific funding for this work.

BT receives research support from the US Department of Veterans Affairs (https://www.newengland.va.gov/research/v1cda/) and the C.G. Swebilius Foundation (https://fconline.foundationcenter.org/fdo-grantmaker-profile/?key=SWEB001).

At this time, please address the following queries:

a)	Please clarify the sources of funding (financial or material support) for your study. List the grants or organizations that supported your study, including funding received from your institution.

*We did not receive any funding (financial or material) for this study.*

b)	State what role the funders took in the study. If the funders had no role in your study, please state: “The funders had no role in study design, data collection and analysis, decision to publish, or preparation of the manuscript.”

*There were no funders for this study.*

c)	If any authors received a salary from any of your funders, please state which authors and which funders.

*While the authors received no specific funding for this work or study, BT receives salary and research support for other projects from the US Department of Veterans Affairs (https://www.newengland.va.gov/research/v1cda/) and the C.G. Swebilius Foundation (*[*https://fconline.foundationcenter.org/fdo-grantmaker-profile/?key=SWEB001*](https://fconline.foundationcenter.org/fdo-grantmaker-profile/?key=SWEB001)*). These funders had no involvement in the study.*

d)	If you did not receive any funding for this study, please state: “The authors received no specific funding for this work.”

*The authors received no specific funding for this work.*

Please include your amended statements within your cover letter; we will change the online submission form on your behalf.

5. In your Data Availability statement, you have not specified where the minimal data set underlying the results described in your manuscript can be found. PLOS defines a study's minimal data set as the underlying data used to reach the conclusions drawn in the manuscript and any additional data required to replicate the reported study findings in their entirety. All PLOS journals require that the minimal data set be made fully available. For more information about our data policy, please see http://journals.plos.org/plosone/s/data-availability.

Upon re-submitting your revised manuscript, please upload your study’s minimal underlying data set as either Supporting Information files or to a stable, public repository and include the relevant URLs, DOIs, or accession numbers within your revised cover letter. For a list of acceptable repositories, please see http://journals.plos.org/plosone/s/data-availability#loc-recommended-repositories. Any potentially identifying patient information must be fully anonymized.

Important: If there are ethical or legal restrictions to sharing your data publicly, please explain these restrictions in detail. Please see our guidelines for more information on what we consider unacceptable restrictions to publicly sharing data: http://journals.plos.org/plosone/s/data-availability#loc-unacceptable-data-access-restrictions. Note that it is not acceptable for the authors to be the sole named individuals responsible for ensuring data access.

We will update your Data Availability statement to reflect the information you provide in your cover letter.

Please review your reference list to ensure that it is complete and correct. If you have cited papers that have been retracted, please include the rationale for doing so in the manuscript text, or remove these references and replace them with relevant current references. Any changes to the reference list should be mentioned in the rebuttal letter that accompanies your revised manuscript. If you need to cite a retracted article, indicate the article’s retracted status in the References list and also include a citation and full reference for the retraction notice.

*Thank you. We have added the following to the box underneath the “Data Availability” section in the submission form:*

*“We have discussed the sharing of our data with the Yale University Privacy Office which made the determination that we are legally and ethically restricted from sharing data because the extent of data poses a risk of re-identification of patients and their HIPAA protected data through deductive disclosure.*

*Susan Bouregy, PhD (susan.buregy@yale.edu) is Yale’s chief privacy officer, and will serve as the contact for the Yale University Privacy Office, to which data requests may be sent.*

*Data cannot be shared publicly because of legal and ethical restrictions regarding patient privacy. Data are available from Yale's chief privacy officer, Susan Bouregy, PhD (susan.buregy@yale.edu), for researchers who meet the criteria for access to confidential data.*

*This was the same case for “Tolchin B, Oladele C, Galusha D, Kashyap N, Showstark M, Bonito J, et al. (2021) Racial disparities in the SOFA score among patients hospitalized with COVID-19. PLoS ONE 16(9): e0257608.* [*https://doi.org/10.1371/journal.pone.0257608*](https://doi.org/10.1371/journal.pone.0257608)*” which used the same dataset and was previously published in your journal.*

*The eICU database is publicly accessible. Pollard, T., Johnson, A., Raffa, J., Celi, L. A., Badawi, O., & Mark, R. (2019). eICU Collaborative Research Database (version 2.0). PhysioNet. https://doi.org/10.13026/C2WM1R.*

1. Is the manuscript technically sound, and do the data support the conclusions?

The manuscript must describe a technically sound piece of scientific research with data that supports the conclusions. Experiments must have been conducted rigorously, with appropriate controls, replication, and sample sizes. The conclusions must be drawn appropriately based on the data presented.

Reviewer #1: Yes

Reviewer #2: Yes

2. Has the statistical analysis been performed appropriately and rigorously?

Reviewer #1: Yes

Reviewer #2: No

3. Have the authors made all data underlying the findings in their manuscript fully available?

The PLOS Data policy requires authors to make all data underlying the findings described in their manuscript fully available without restriction, with rare exception (please refer to the Data Availability Statement in the manuscript PDF file). The data should be provided as part of the manuscript or its supporting information, or deposited to a public repository. For example, in addition to summary statistics, the data points behind means, medians and variance measures should be available. If there are restrictions on publicly sharing data—e.g. participant privacy or use of data from a third party—those must be specified.

Reviewer #1: Yes

Reviewer #2: Yes

4. Is the manuscript presented in an intelligible fashion and written in standard English?

PLOS ONE does not copyedit accepted manuscripts, so the language in submitted articles must be clear, correct, and unambiguous. Any typographical or grammatical errors should be corrected at revision, so please note any specific errors here.

Reviewer #1: Yes

Reviewer #2: Yes

5. Review Comments to the Author

Please use the space provided to explain your answers to the questions above. You may also include additional comments for the author, including concerns about dual publication, research ethics, or publication ethics. (Please upload your review as an attachment if it exceeds 20,000 characters)

Reviewer #1: The authors performed a retrospective study to evaluate whether SOFA score is desirable in predicting in-hospital mortality of COVID-19 patients and compared it with simple use of age. The authors concluded age is better than SOFA score. Although using age for predicting mortality of COVID-19 patients is still far from perfect, the information derived from this study is very important to the field. The methods used in the study was straightforward, I have no criticism on the analysis.

*We thank reviewer one for this positive feedback about our manuscript and taking the time to review it.*

Reviewer #2: It is a very interesting study. "Caution is needed when applying an established disease severity index model to a new illness." A few minor issues warrant attention:

1.In the "methods" section of the article, it is stated: "Age in years at time of admission and the maximum SOFA score recorded in the first 24 hours after admission were each used as predictor variables in separate univariate logistic regression models for the binary outcome of in-hospital mortality. Each model was fit to a sample of 60% of the respective cohorts." However, in the “results”section, relevant displays of these outcomes were not observed.

*We additionally thank this reviewer for their interest in our manuscript.*

*We would like to take this opportunity to provide clarification for the “relevant displays” requested by the reviewer. In this work, we develop univariate logistic regression models of COVID-19 mortality risk. In order to assess the accuracy of the univariate logistic regression model, we use area under the receiver operating characteristic curve (AU-ROC), area under the precision-recall curves (AU-PRC). These are found in Figure 2. Based on this reviewer’s feedback, we now include calibration curves which are important to understanding the clinical utility of a prognostic model. These additional analyses are reflected in the methods section and the calibration curve figures are included as the new Supplemental Figure 1 with observed probability of event (mortality) on the Y axis and predicted event rate (mortality) on the X axis.*[*[1]*](https://paperpile.com/c/H4OPue/Zqun)

2.It is imperative to present the results where age, after adjusting for common confounding factors (comorbidities, creatinine, total bilirubin, INR, etc.) in both cohorts, is independently associated with prognosis. Additionally, for the SOFA score, the prognostic predictive value after adjusting for confounding factors should also be demonstrated.

*We thank this reviewer for this feedback. Our goal was to describe the utility of these measures as strictly univariate screening tools, rather than in a more complex algorithm. Moreover, two proposed confounders (creatinine, bilirubin) are components of SOFA. We are unsure of how we can adequately control for these potential confounders when they are part of the exposure.*

3.Obviously, in this study, the author aims to assess the discriminative performance of SOFA scores on patient mortality and compare it with age. So, in addition to comparing the differences in AUC (statistical test results were not provided in the article), a comprehensive judgment needs to be made by combining NRI and IDI.

*To these author’s understanding, NRI and IDI refer to the change in performance once a second test is added to a baseline measure.*[*[2]*](https://paperpile.com/c/H4OPue/nPCs) *In our study, there is no baseline measure, but instead a head-to-head comparison of two univariate measures (age and SOFA score). We instead added a decision curve analysis (DCA) as a new Figure 3 to specifically address tension related to clinical decision making.*[*[3]*](https://paperpile.com/c/H4OPue/tRZn) *Consistent with earlier findings in our paper, the age based prediction model has a higher net benefit than the SOFA Score based model across a large range of threshold probabilities in the COVID cohort, while the opposite phenomena is observed in the eICU (non-COVID) cohort.*

1.	[Kuhn M, Vaughan D, Ruiz E. probably: Tools for Post-Processing Class Probability Estimates. 2023. Available:](http://paperpile.com/b/H4OPue/Zqun) [https://github.com/tidymodels/probably/, https://probably.tidymodels.org}](https://github.com/tidymodels/probably/,%20https://probably.tidymodels.org%7D)

2.	[Hilden J. Commentary: On NRI, IDI, and “good-looking” statistics with nothing underneath. Epidemiology . 2014. pp. 265–267. doi:](http://paperpile.com/b/H4OPue/nPCs)[10.1097/EDE.0000000000000063](http://dx.doi.org/10.1097/EDE.0000000000000063)

3.	[Fitzgerald M, Saville BR, Lewis RJ. Decision curve analysis. JAMA: the journal of the American Medical Association. 2015. pp. 409–410. doi:](http://paperpile.com/b/H4OPue/tRZn)[10.1001/jama.2015.37](http://dx.doi.org/10.1001/jama.2015.37)
