# Supplementary material for: SOFA score performs worse than age for predicting mortality in patients with COVID-19
Source: PLoS One. 2024 May 17;19(5):e0301013. doi: 10.1371/journal.pone.0301013 (PMC11101117; doi:10.1371/journal.pone.0301013)
Supplement: Attachment — Submitted filename: Response to Reviewer Comments.pdf [file pone.0301013.s005.pdf]

Response to Reviewer Comments:

Reviewer #1: Few major comments to address:
1. Although the data can not be presented, supplementary items can benefit from consort diagrams of both COVID and eICU cohort derivation.

- *We did not feel that a consort diagram would add significant value to this publication as the COVID-19 cohort has been previously described in two prior Plos One publications (*Tolchin B, Oladele C, Galusha D, Kashyap N, Showstark M, et al. (2021) Racial disparities in the SOFA score among patients hospitalized with COVID-19. PLOS ONE 16(9): e0257608. https://doi.org/10.1371/journal.pone.0257608 *and* Roy S, Showstark M, Tolchin B, Kashyap N, Bonito J, et al. (2021) The potential impact of triage protocols on racial disparities in clinical outcomes among COVID-positive patients in a large academic healthcare system. PLOS ONE 16(9): e0256763. https://doi.org/10.1371/journal.pone.0256763) *the latter of which forwent including a consort diagram. The eICU database has been described in innumerable publications.*

2. The covid-19 cohort has significant difference in ICU stay and some racial distribution. Also it is extremely small in size compared to eICU cohort. eICU cohort is far more advantageous in the fact that 100% of patients are ICU patients, and SOFA was indeed designed for such purpose. The comparison may not be 'apples to apples' in this case. Authors may look into cohorts that can be more comparative in this regard.

- *To address the difference in ICU status, we also did a sub-group analysis restricted to ICU patients in the COVID-19 cohort. As described in the text, the COVID-19 ICU subgroup had similar results to the larger COVID-19 cohort. While a larger cohort size is always useful, the purpose of using the eICU cohort was to inquire as to whether any findings are unique to patients with COVID 19.*
- *Our sample size was large of enough and our study was adequately powered to demonstrate our key findings.*

3. in case of age and SOFA scores, it would be more meaningful to see statistics like medians, IQRs, etc to have better understanding of feature distribution. Feature distribution can play a significant role in Logistic regression method used.

- *A much-appreciated point. We have updated our manuscript to include median and IQRs for age and SOFA score.*

4. Apart from only reporting AUROC and AUPRC scores for Black and White population, there are model bias analysis that can be performed. Authors may consider a proper model bias testing rather than only providing model accuracy scores.

- *It is unclear which specific model bias tests reviewer #1 is referring to. Furthermore, we are less concerned with model bias as our key conclusion is that SOFA score performs*

poorly for mortality prediction in patients with COVID-19 and should not be used for crisis standards of care. We do not suggest using age alone as a model for resource allocation, but rather compare SOFA score to age to demonstrate just how little value the SOFA score has for this purpose.

5. It has been long established by now that age is indeed a major factor in determining mortality of older COVID-19 patient cohort. Authors may want to consider what new significant information is being presented through this work.

- *Indeed, patient age has now been established as highly correlated with mortality in COVID-19 patients. The fact age performed better than SOFA score in both Raschke et al. and our analyses raises considerable concern that SOFA score continues to feature prominently in protocols for crisis standards of care. While this study is a confirmatory study, it addresses questions and limitations of the study by Raschke et al. such as small sample size and patients from a single geographic area. Our study includes over 4 times the number of patients from the study by Raschke et al., all from a different geographic area. We think our study demonstrates that prior findings are likely externally valid as well as unlikely to be coincidental or spurious. Confirmatory studies such as ours are often needed to motivate policy change.*

Reviewer #2: The authors present a well timed article to help the medical community discern the utility Sequential Organ Failure Assessment(SOFA) as a predictor for in-hospital mortality of COVID19 patients. The authors find that SOFA scores perform worse than age in univariate anaysis to predict COVID 19 mortality. The authors also contrast the above mentioned finding with their conclusions from univariate analysis of non-COVID patients from the eICU database. For the eICU database the results show that SOFA is a better predictor than age for patient morality.
The findings have significant impact for the resource allocation decisions for the hospital administrators. The paper also cautions the medical community to the fact that well used morbidity discriminators such as SOFA may not be transferrable to a new disease.
Comments on each section
Introduction - The introduction section is satisfactory and clearly explains the aim of the research.

- *Thank you.*

Methods - The sections explains the methods with clarity. The section states that admissions SOFA was used as independent varibale. The choice of model in logistic regression is simple but acceptable . Instead of using just SOFA, it may have been useful to anlyse data using delta SOFA over twenty four hour period. It may also be useful to study the prediction capability of SOFA within various age groups.

- *Thank you for these insightful observations. We used max SOFA score because that was the measure used in most of the resource triage protocols we examined (including the YNHH protocol).*
- *It would be an interesting area for future work to examine whether delta SOFA score has any value for mortality prediction as well as to evaluate whether SOFA score performs better in certain patient age groups. Nonetheless, for the purposes for resource allocation we would recommend that a COVID-19 specific mortality prediction scoring model be used.*

Results - The section is clear and concise. Figure 1 has some dataset in brown, but the legend does not clearly state what that data refers to? Other figures are clear and appropriate.

- *Regarding figure 1, the data that appear in brown reflect the areas of overlap between the eICU data (blue) and COVID-19 data (orange).*

Discussion - The authors present an honest discussion to the limitations of the study as well the limited understanding of why age is a better morbidity predictor than SOFA for COVID19 patients. I urge the authors to consider the dynamical evolution of symptoms for COVID19 in a given patient. If a disease causes rapid deterioration, then admittance SOFA may not be able to predict patient outcome but a delta SOFA over twenty hours maybe a better predictor? For diseases that cause a slower decline in health (as is most likely the case for many eICU patients) the state of the organs at admittance is likely to be a better discriminator. Also for an apples to apples comparison, if possible eICU dataset can be parsed for admittance due to influenza (respiratory disease causing mortality in older populations) and then a comparative study be done with COVID19 dataset.

- *Thank you for your thoughtful insights. We agree that a delta SOFA score may perform better than max SOFA score in the first 24 hours after admission. However, it is difficult to choose which 24-hour period to calculate the delta SOFA score, as is astutely mentioned above, COVID can cause a rapid deterioration later in the hospital course. We chose max SOFA score because that was what we saw most prominently used in protocols for crisis standards of care. This is likely under the assumption that resource allocation would take place within the first 24 hours of admission.*
- *It would be interesting to conduct a similar analysis on eICU dataset restricted to patients with influenza, however, how to identify this patient population may be deceptively difficult. ICD codes may not reflect the actual active clinical diagnosis (*Quan H, Li B, Saunders LD, Parsons GA, Nilsson CI, Alibhai A, Ghali WA; IMECCHI Investigators. Assessing validity of ICD-9-CM and ICD-10 administrative data in recording clinical conditions in a unique dually coded database. Health Serv Res. 2008 Aug;43(4):1424-41. doi: 10.1111/j.1475-6773.2007.00822.x. PMID: 18756617; PMCID: PMC2517283*), natural language processing can be fraught with misclassification, and often influenza patients have a related, but different diagnosis such as "respiratory distress" or "dehydration" as the reason for admission.*

I congratulate the authors for studying a pertinent problem and at short notice, adding to the body of knowledge that can help navigate the medical care for this global pandemic.
